# The National Pension Fund and Workers.—II

**Published:** 1890-11-29

**Authors:** 


### Passing Topics.

### THE NATIONAL PENSION FUND AND WORKERS.—II.

In our last we set forth, in a few sentences, the general scope of this society and the advantages it offers to its policy-holders. We have now to consider the claims of hospital officials to fair, and even liberal treatment in regard to retiring allowances; and first, let us say, it strikes us as eminently satisfactory that the operations of the National Pension Fund have not been limited to nurses, but have been extended to embrace hospital officials of every grade. Even if this extension should not ultimately enlarge the popularity of the Fund to the degree we anticipate it will, the mere fact that the society is comprehensive adds much to its moral recommendations, and cannot fail to increase its stability. Except in a few special instances, all hospital workers alike are but poorly remunerated, and command but scanty opportunities to make provision for old age without some such facilities as are offered by this society.

Apart from the moral obligation which rests upon employers, there is a distinct economical advantage to themselves, whether they be individuals, companies, or institutions, when they succeed in arousing in their officials something more than a merely day by day interest in the concerns they have to deal with. In hospital life few things are more pleasing than the solicitude shown by a large proportion of workers for the well-being and reputation of the particular institutions with which they are connected, and nothing is better calculated to foster this feeling, where it is wanting, than a means of accustoming the mind of the worker to the thought that he is not only presently identified with the institution, but that his future relation to it is assured, and that long and faithful service will not go unrewarded.

Men and women who devote the best of life's powers to the promotion of interests not their own, and to the exclusion of all possibility of providing against the rainy days which inevitably come to all, are clearly entitled to something when they can work no more; and it is much to be desired that the terms of an engagement should invariably include an understanding as to a retiring allowance. Reasonable outlay under this head might well form a part of every hospital's normal expenditure, and no man of business would be likely to call it in question. Of course, a fair length of service must be insisted upon before any claim for a pension could be entertained, and if that were reckoned at a minimum of, say, twenty years, there would be no need for the most timorous financier to become alarmed at the prospect.

Well, it is some such provision as this that the National Fund is prepared to make upon the lowest terms consistent with security, and to add besides those extra advantages enumerated in our last. Very properly, the managers look to the worker to make the first move, but recognising the validity of such arguments as we have ventured upon, they do not propose to leave him, unaided, to his own resources. On the contrary, the very important suggestion is made that a part of the premium should be borne by the institution he serves, which thus, by a comparatively small and regular payment, would become insured against a heavy outlay at some future time.

How this proposal is regarded by the hospitals is very well shown by the favourable reception accorded to it in several influential quarters. The authorities of the London Hospital, Guy's, King's College, St. Mary's, the Mildmay Institution, the Seamen's Hospital, and many other metropolitan and provincial hospital and nursing societies have already affiliated themselves to the fund, and have engaged to pay one-half the premiums of every nurse in their employ, upon certain conditions. To the remaining hospitals the best advice we can offer is to go and do likewise. Agricola.

